# Oxidation of Various Kraft Lignins with a Bacterial Laccase Enzyme

**DOI:** 10.3390/ijms222313161

**Published:** 2021-12-06

**Authors:** Sebastian A. Mayr, Raditya Subagia, Renate Weiss, Nikolaus Schwaiger, Hedda K. Weber, Johannes Leitner, Doris Ribitsch, Gibson S. Nyanhongo, Georg M. Guebitz

**Affiliations:** 1 Institute of Environmental Biotechnology, University of Natural Resources and Life Sciences (BOKU), Konrad Lorenz Strasse 20, 3430 Tulln, Austria; sebastian.mayr@boku.ac.at (S.A.M.); doris.ribitsch@boku.ac.at (D.R.); g.nyanhongo@boku.ac.at (G.S.N.); guebitz@boku.ac.at (G.M.G.); 2 Austrian Centre for Industrial Biotechnology (ACIB), Konrad Lorenz Strasse 20, 3430 Tulln, Austria; radityasubagia@acib.at; 3 Sappi Paper Holding GmbH, Brucker Strasse 21, 8101 Gratkorn, Austria; Nikolaus.Schwaiger@sappi.com (N.S.); hedda.weber@sappi.com (H.K.W.); 4 Mondi Aktiengesellschaft, Marxergasse 4A, 1030 Vienna, Austria; johannes.Leitner@mondigroup.com

**Keywords:** kraft lignin, lignosulfonate, CotA, laccase, oxidation, purity, isolation, precipitation

## Abstract

Modification of kraft lignin (KL), traditionally uses harsh and energy-demanding physical and chemical processes. In this study, the potential of the bacterial laccase CotA (spore coating protein A) for oxidation of KL under mild conditions was assessed. Thereby, the efficiency of CotA to oxidize both softwood and hardwood KL of varying purity at alkaline conditions was examined. For the respective type of wood, the highest oxidation activity by CotA was determined for the medium ash content softwood KL (MA_S) and the medium ash content hardwood KL (MA_H), respectively. By an up to 95% decrease in fluorescence and up to 65% in phenol content coupling of the structural lignin units was indicated. These results correlated with an increase in viscosity and molecular weight, which increased nearly 2 and 20-fold for MA_H and about 1.3 and 6.0-fold for MA_S, respectively. Thus, this study confirms that the CotA laccase can oxidize a variety of KL at alkaline conditions, while the origin and purity of KL were found to have a major impact on the efficiency of oxidation. Under the herein tested conditions, it was observed that the MA_H KL showed the highest susceptibility to CotA oxidation when compared to the other hardwood KLs and the softwood KLs. Therefore, this could be a viable method to produce sustainable resins and adhesives.

## 1. Introduction

**Figure 1:**
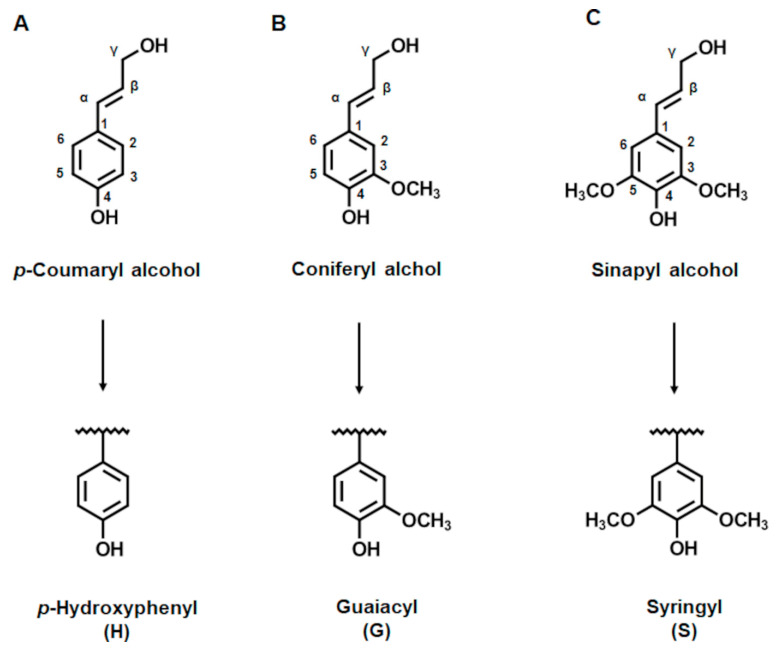
Structures of the monolignols: *p*-coumaryl alcohol, which has no methoxy group (**A**), coniferyl alcohol, which has one methoxy group at position C3 (**B**) and sinapyl alcohol, which has two methoxy groups at positions C3 and C5 (**C**). The monolignols represent the precursors for the structural units in lignin. Below the elemental monomers *p*-Hydroxyphenyl (H), Guaiacyl (G) and Syringyl (S) are depicted.

Due to the increasing awareness of mankind on the negative impacts of the use of fossil-based resources on climate change and environmental pollution, great efforts are being made to find sustainable alternatives to replace their use [1,2]. Lignin is such a sustainable raw material presenting the second most abundant natural biopolymer. Together with cellulose and hemicellulose, lignin forms the biomass of plants [3]. Cellulose and hemicellulose are used to produce high-quality paper or bioethanol amongst many other products and thus are considered high-value materials. Lignin, on the other hand, is regarded as low-value material and is mostly burned to recover some of the energy needed in the respective facilities [4,5]. Lignin is a highly recalcitrant biopolymer with a complex and heterogeneous structure, presenting the biggest obstacle to its use. While simultaneously, the value of lignin lies buried within its structure as it comprises mainly aromatic compounds [6,7]. Lignin is composed of three repetitive structural units, the so-called monolignols. These are presented by *p*-coumaryl alcohol, sinapyl alcohol and coniferyl alcohol. When these monolignols are incorporated into the growing lignin polymer they form *p*-hydroxyphenyl (H), syringyl (S) and guaiacyl (G) units, respectively. All these units contain phenol groups and differ only in the number of methoxy groups attached to the aromatic ring. H has none, G one at C3 position, and S two at C3 and C5 (Figure 1A–C). The composition of lignin varies within the respective type of biomass. Grasses, for example, consist mainly of H and G units while in woods G and S units are predominant. The abundance and the ratio of these monolignols determine the reactivity of the respective lignin and can be defined, e.g., by calculating the S/G ratio using various structural analyses, like Fourier Transform Infrared (FTIR) or Nuclear Magnetic Resonance (NMR) spectroscopy [3,6].

Common strategies for adding value to lignin comprise depolymerization, polymerization and modification [8]. Regarding depolymerization, lignin is broken down into smaller molecules, which can then be used as raw material for the production of platform chemicals, like vanillin [9]. Further polymerization of lignin, on the other hand, was reported to increase dispersing properties, making the polymers suitable for applications in binders, concretes, resins or coatings [10,11]. The third approach to upgrading lignin involves predominantly chemical modification, enzymatic modification has been described mainly for lignosulfonates (LS) and native lignin [12,13,14]. The resulting functional materials conveying antimicrobial or antioxidant activity can be used in adhesives, as graft co-polymers or in biomedical applications [15,16,17].

The structure of isolated lignin varies greatly and depends on the kind of biomass, either softwood or hardwood, and the type of isolation process used [6]. Removal of lignin by sulfite pulping results in LS, which are water-soluble, of high molecular weight but with a lower concentration in phenolic groups. On the other hand, kraft pulping produces so-called kraft lignin (KL), which are insoluble in tap water, of low molecular weight but with a higher content in phenolic groups [6,7,18]. Nowadays, kraft pulping is the dominant industrial pulping process, making KL the most available type of isolated lignin [4].

During kraft pulping, wood chips are cooked between 150–170 °C for several hours in an aqueous solution of NaOH and NaS_2_ (white liquor) causing separation of biomass into its single compounds. Under these harsh conditions, the actually recalcitrant lignin is dissolved, indicated by a color change of the liquor from white to black [10]. The pH of this black liquor typically ranges from 13 to 14, where the functional groups of lignin, such as methoxy, hydroxyl, carbonyl and carboxyl groups, are present in their deprotonated and thus dissolved form, stabilized through electrostatic repulsion [19]. However, the black liquor not only contains lignin but also some impurities like residual cellulose and hemicellulose, inorganics and other compounds, simply referred to as ash hereinafter.

One possible way to get rid of ash is the precipitation of lignin from the black liquor by acidification. This process is commercially applied and known as the LignoBoost process. Presenting a low-cost process resulting in high purity and high yields of the final isolated KL [20,21].

In contrast to the industrial delignification processes, in nature, plants and fungi produce enzymes to metabolize or degrade lignin, like peroxidases or laccases [22]. Unlike peroxidases, which require hydrogen peroxide as co-substrate, laccases only need molecular oxygen, making the latter more attractive for industrial uses [23]. Laccases (EC 1.10.3.2, *p*-diphenol:dioxygen oxidoreductase) are multi-copper proteins that generate radicals by oxidizing phenols and aliphatic or aromatic amines while reducing molecular oxygen to water [24,25]. The substrate range of the respective laccase depends on its redox potential. Enzymes with a low redox potential (0.4 V and below) react only with the phenolic structures in lignin, while high redox potential laccases (above 0.4 V) can also interact with other structural residues, like aliphatic or aromatic amines [26,27]. Nowadays, besides in fungi and plants, enzymes showing laccase activity were also isolated from bacteria and insects [22,24,25].

In order to use KL for high-value applications, structural modifications are needed, either to increase the homogeneity and availability of reactive groups or to tailor new properties. Most of these modifications are conventionally achieved through chemical reactions, which require the use of expensive and sometimes environmentally harmful organic solvents and are conducted under harsh reaction conditions. Thus, running these modification processes with lower waste accumulation and at milder conditions would be beneficial. A possible way to achieve this is presented by the use of biocatalysts, such as laccases [23,28]. However, the water insolubility of KL, and most of the other technical lignins, turned out to hamper the shift from chemical to enzymatic modification [6].

Nevertheless, by now, several attempts were made to enzymatically modify KL. Moya et al. showed that both fungal and bacterial laccases can be used to modify lignin under alkaline conditions [29]. Gouveia et al. enzymatically polymerized KL isolated from black liquor with a low redox potential fungal laccase with and without the use of mediators. Thereby, it turned out that the use of mediators did not improve the results significantly [30,31]. A fungal laccase with a high redox potential was used to investigate the possibility to polymerize diverse technical lignins at an acidic pH by Mattinen et al. with the outcome that polymerization occurs [32]. Recently, Choolaei et al. tested the possibility of a bacterial laccase to oxidize model substrates and sulfonated KL (a chemically modified KL making it water-soluble) at neutral pH and found that the bacterial laccase showed the ability to either polymerize or depolymerize the therein investigated samples by oxidation [33]. The same was found for a laccase of bacterial origin genetically modified in a way to be active also at high alkaline pH of 10.5 and even higher [34].

Regarding the use of biocatalysts for lignin modification, the use of LS turned out to be beneficial, due to its water solubility. Thus, a process for laccase catalyzed oxidation of LS without the need of mediators but with a steady oxygen supply was recently developed by our group, allowing extensive polymerization of LS [35,36]. This resulted in polymers that showed a better dispersibility and were found to be applicable in coating formulations or as nanoparticles for fertilizers [37,38]. This process is well implemented for LS, allowing the use of fungal laccases due to the water solubility of LS. Most of the fungal laccases are active under acidic to neutral pH conditions, at moderate temperatures and have low to high redox potentials (0.4 to 0.8 V) [22,39]. This renders them as well suited for the enzymatic modification of LS but unfortunately, they cannot be applied with KL directly. This is because, at these respective pH values, KL is not soluble, as it starts to dissolve at a pH of 8 and higher, with further increasing solubility with increasing pH [28,40,41]. A promising way to achieve enzymatic polymerization of KL can be the use of laccases of bacterial origin, which are unlike fungal laccases also active at higher pH values and aside from that also show thermostability and higher salt tolerance, making their use in industrial processes beneficial. However, besides these advantages, bacterial laccases also come along with some drawbacks, like low yields and activity, difficult purification and lower redox potentials (generally below 0.4 V) when compared to fungal laccases [42,43]. One of these bacterial enzymes showing laccase activity is presented by the coating protein A (CotA) as found in *Bacillus subtilis* [44,45,46].

Most of the above-mentioned studies were carried out at an acidic pH, due to the pH optimum of the used fungal laccases. In this milieu, it is necessary to dissolve KL either in organic solvents or ionic liquids and transfer it into the respective buffer solution for the reaction [29,32]. Only a few studies were conducted where the oxidation capacity of laccases on lignin was investigated at alkaline conditions by using either fungal [30,31] or bacterial laccases [33,34]. None of them applied a steady oxygen supply as was the case in this study, which was found to lead to higher polymerization rates for LS [36].

In the present work, the ability of the CotA laccase to oxidize KL of different origins and purity at alkaline conditions without the use of mediators but with a steady external oxygen supply was investigated. The progress of the reaction was followed by monitoring the changes in fluorescence, phenol content, viscosity, molecular weight and oxygen consumption. Where fluorescence and phenol content decrease upon enzymatic oxidation while viscosity and molecular weight increase due to subsequent polymerization. Finally, a comparison between the outcomes of the enzymatic oxidation process of LS with a laccase of fungal origin and the herein investigated KL process with the bacterial laccase was made.

## 2. Results and Discussion

In contrast to most fungal laccases, those of bacterial origin were found to be active also under alkaline pH conditions, e.g., at pH 8, like the herein used CotA laccase. Thus, CotA is a very promising candidate to investigate its ability to oxidize kraft lignin (KL) of different origins and purity under alkaline conditions without the use of mediators but with a steady oxygen supply as a co-substrate.

In a first attempt, to determine whether the CotA enzyme was able to react with KL at all, the various KL were tested for their reactivity as received (unfractionated). These first experiments with the unfractionated KL were run at 30 °C because this temperature was found to be the optimum for enzymatic oxidation of LS [47]. Since the ability of CotA to oxidize KL was already demonstrated by these first experiments, in the next step, the effects of a more homogeneous molecular weight distribution, achieved through membrane filtration (retentate), and of a higher temperature on the oxidation of KL were determined. Furthermore, the best results obtained herein were compared to the results achieved with the lignosulfonate (LS) process.

### 2.1. Characterization of the KL Samples

The received KL samples were characterized in terms of initial pH, molecular weight, S/G ratio, dispersity (Ð) and total phenol content.

The samples precipitated at higher pH are of high ash content (HA), while those precipitated at lower pH are of medium (MA) to low (LA) ash content. This is due to the degree of protonation, which is lower at higher pH values leading to a fully protonated KL at pH 3 [19,48,49].

It is known that the grade of purity and the size of the KL molecules are influenced by the precipitation pH used [7,19,50,51]. During kraft pulping the lignin molecules are degraded into smaller oligomers of low molecular weights between 2 to 200 kDa [18,52]. In general, softwood KL are known to be of higher molecular weight than hardwood KL, which was confirmed in this study (Table 1). This is due to the predominance of G units in softwood KL, which possesses only one methoxy group at position C3 and a reactive site at position C5 (Figure 1B). Hence, tending to form branched structures, which leads to overall bigger molecules. Whereas, in hardwood KL, the abundance of both G and S units, where both C3 and C5 positions are occupied in the latter by methoxy groups (Figure 1C), leads to the formation of primarily linear structures and thus results generally in smaller molecules [53].

During the precipitation process, lignin molecules of different sizes are formed. The molecular weight distribution, known as Ð, is determined by forming the ratio between the high molecular weight (weighted average; Mw) to the low molecular weight fraction (numbered average; Mn). Herein, it was found that Ð became smaller with decreasing pH (Table 1).

Amongst others, the determination of the S/G ratio allows to conclude about the susceptibility towards enzymatic oxidation of lignin and was reported to be higher for the hardwood lignins, due to the abundance of both S and G units [54]. Further, it was found that for hardwood lignins a lower S/G ratio indicated a higher susceptibility for enzymatic oxidation [30,31].

Higher content in phenolic groups leads to higher reactivity, while aliphatic OH-groups are supposed to have a lower reactivity, due to the mainly occurring condensation reactions (formation of C-C bonds) [21]. Generally, KL comprises a higher phenolic OH-group content when compared to other technical lignins, due to extensive cleavage of β-aryl bonds during cooking [52]. This was found also for the received samples, with the hardwood KL showing higher phenol content than the softwood KL (Table 1).

In general, the hardwood KL (H) showed higher S/G ratios and higher concentrations in total phenols, while the softwood KL (S) showed higher initial molecular weights and Ð (Table 1). Further, it was observed that with decreasing pH and thus increasing purity, the ash content, Ð and molecular weight decreased, while total phenol content increased, which is in accordance with literature [48].

These trends were found for all herein investigated samples, except for the medium ash softwood KL (MA_S), which showed a higher initial molecular weight, Ð and phenol content than the other softwood samples. However, especially with softwood KL, this unsteady behavior was observed earlier [48].

### 2.2. Oxidation of Diverse KL Samples by the CotA Laccase

In a first attempt, the received KL samples were oxidized by CotA as received (unfractionated). In order to determine the efficiency of CotA oxidation the changes in fluorescence, viscosity, phenol content, molecular weight and oxygen consumption were monitored.

**Figure 2:**
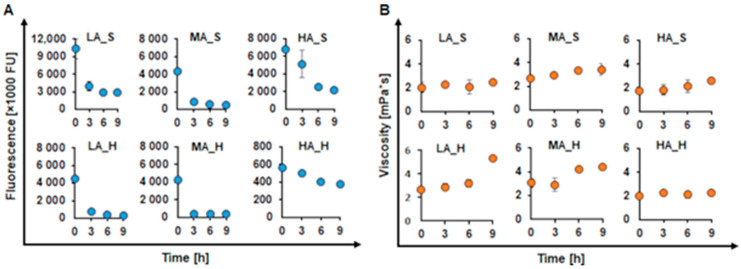
Changes in fluorescence (**A**) and viscosity (**B**) of softwood (S) and hardwood (H) for the unfractionated fractions of the kraft lignin (KL) samples with different ash content (low (LA), medium (MA) and high (HA)) during oxidation with CotA (blue and orange dots, respectively). All reactions were done at pH 9 with 1 U/mL CotA at 30 °C and with a steady oxygen supply.

All unfractionated KL samples showed a decrease in fluorescence throughout the reaction. The unfractionated low ash and high ash softwood KL (LA_S and HA_S) were found to decrease in fluorescence by 70%, while the medium ash softwood KL (MA_S) decreased in fluorescence by 90%, with the lowest decrease found for HA_S. The unfractionated low and medium hardwood KL (LA_H and MA_H) showed a decrease in fluorescence by 90%, while the high ash hardwood KL (HA_H) decreased in fluorescence only by 30%. Thus, not only did the HA sample again show the lowest decrease in fluorescence, but in the case of hardwood, it also was lower by a power of ten (Figure 2A).

Fluorescence is an internal property of lignin, caused by stilbene-, carbonyl-, biphenyl- and phenylcoumarin-groups, which are present in the structure of lignin [55,56]. The addition of laccase to lignin initiates oxidation of the phenolic OH groups of lignin leading to the formation of highly reactive phenoxy radicals. These formed radicals can cross-react with each other leading to the formation of new carbon-carbon (C-C) or ether bonds (β-O-4) [57]. The formation of these new bonds causes a disturbance in the structure of these functional fluorescent groups, leading to a loss in fluorescence intensity [36,56]. The presence of ash is known to lead to quenching of fluorescence, which would explain the low values for fluorescence observed within HA_H [58,59]. This trend was found to be true at least for hardwood, in which the higher purified samples showed higher fluorescence. On the contrary, the softwood KL samples did not show a clear trend in fluorescence upon enzymatic oxidation, which was already observed for MA_S KL before (Table 1). These findings were further confirmed by the changes in viscosity, which were found to increase for almost all samples upon oxidation by CotA. The unfractionated MA_S and HA_S increased in viscosity by 1.3 and 1.5-fold, respectively, while the unfractionated LA_S did not show any change in viscosity. Interestingly, in contrast to the softwood KL, for the hardwood KL, the one showing no change in viscosity was the HA_H, while the LA_H and MA_H increased viscosity by 2.0- and 1.4-fold, respectively (Figure 2B).

The lack in reactivity of LA_S (Indulin AT) may be due to the different isolation processes used to produce Indulin AT KL and LignoBoost KL resulting in a lower amount of aromatic hydroxyl groups for the former, thus suggesting a lower reactivity [7,60,61,62]. This is also in concordance with the lower phenol content observed in LA_S (Table 1). Whereas for HA_H, both the high ash content and the presence of low molecular weight phenolics could be responsible for the no change in viscosity detected. While inorganic molecules might inactivate the enzyme, low molecular weight phenolics could be a competitive substrate for the enzyme “keeping the enzyme busy”. Both the unfractionated LA_S and HA_H showed decreasing fluorescence upon enzymatic oxidation but no changes in viscosity, indicating their lack in reactivity. However, the reactivity of HA_H can probably be further increased by membrane filtration and was thus investigated further.

Besides acidification, filtration is a frequently used fractionation method for lignin. Upon filtration, the homogeneity of lignin can be further increased by removing, e.g., low molecular weight inhibitors and or competitive laccase substrates, which subsequently leads to the higher reactivity of lignin [21]. Thus, the retentate fractions were further investigated for their ability to get oxidized by the CotA laccase. Whereas the filtrate fractions, containing an increased concentration of ash, were not expected to be oxidizable and thus were not considered for the further experiments. For this set of experiments, the oxidation was carried out at 50 °C since laccases of bacterial origin are known to be thermostable [42,43].

**Figure 3:**
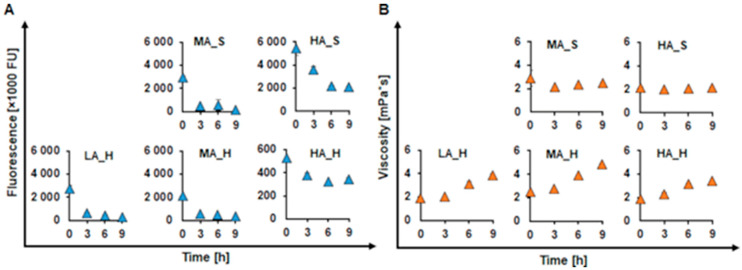
Changes in fluorescence (**A**) and viscosity (**B**) of softwood (S) and hardwood (H) retentate fractions of the KL samples with different ash content (low (LA), medium (MA) and high (HA)) during oxidation with CotA (blue and orange triangles, respectively). All reactions were done at pH 9 with 1 U/mL CotA at 50 °C and with a steady oxygen supply. The LA_S sample was found unreactive in the previous experiments and thus, it was not considered for the fractionation any further.

A decrease in fluorescence was also found for all examined retentate KL. In softwoods, the most pronounced decrease was found for MA_S, with a decrease of 95%, while HA_S decreased only by 62%. The retentate hardwoods showed a decrease in fluorescence by 89% for the LA_H, 84% for the MA_H and only 34% for the HA_H. Generally, when compared to the unfractionated KL, all retentate KL fractions showed a lower initial fluorescence. (Figure 3A). In terms of viscosity, no increase was found for enzymatically oxidized retentate softwood KL (MA_S and HA_S), while the viscosity for the retentate hardwood KL increased up to 2.0-fold (LA_H and MA_H). Interestingly, also the HA_H retentate fraction showed a 1.8-fold increase in viscosity (Figure 3B). Upon fractionation not only the homogeneity of the starting molecular weight was increased but also the content of low molecular weight phenolics was reduced and the ash was accumulated in the filtrate fraction, resulting in a clear viscosity increase upon enzymatic oxidation of the HA_H retentate fraction. The generally better results for the hardwood samples can be explained by their structure. As mentioned before hardwoods contain both S and G units. The latter has two electron-donating methoxy groups in their structure, leading to an increased occurrence of β-O-4 bonds, which are known to be more reactive than the C-C bonds, predominant within the S units in softwoods, which make hardwoods easily oxidizable by laccase [21,31].

Based on the findings made so far, the highest activity of CotA for both types of wood was found for unfractionated MA_S and MA_H KL, respectively. Thus, only these two samples were further investigated related to phenol content and molecular weight changes.

**Figure 4:**
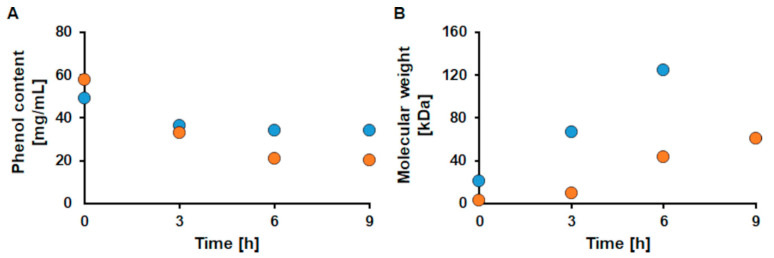
Changes in phenol content (**A**) and molecular weight (**B**) for the unfractionated medium ash softwood (MA_S) KL (blue dots) and the unfractionated medium ash hardwood (MA_H) KL (orange dots) during oxidation with CotA. The reactions were done at pH 9 with 1 U/mL CotA at 30 °C and with a steady oxygen supply.

The content of phenol groups in lignins decreases upon the formation of the radicals, while the molecular weight increases due to radical-induced cross-linking. Enzymatic oxidation by CotA lead to a decrease of the phenol content of MA_S and MA_H by 30% and 65%, respectively (Figure 4A). Concomitantly a 6.0-fold increase, to a molecular weight of 124.7 kDa, was seen for MA_S after 6 h of reaction (filtration of the sample taken after 9 h was not possible anymore, supposedly due to the high molecular weight), while MA_H increased molecular weight even by 19.2-fold after 9 h of reaction to 60.6 kDa (Figure 4B).

The decrease in phenol content was expected upon laccase oxidation. The formation of new bonds between the formed phenol radicals leads to a lower abundance of free phenol groups. The decrease was higher for MA_H than for MA_S, which again confirms the higher reactivity of hardwood as discussed above. The extended increase in molecular weight did not fully correlate to the increase of viscosity seen for these hardwood and softwood samples, which may be due to the fact that hardwood lignin mainly forms linear polymers [21]. Additionally, in former studies, it was found that especially for hardwoods, smaller initial molecules led to the biggest increases in molecular weight [34]. Softwoods are known to form higher condensed molecules (C-C bonds), leading to a lower reactivity. Furthermore, mass transfer and accessibility of the phenol residues are expected to be better when the molecules are smaller, which would herein be the case for the hardwoods [21,30,31,33,34]. In similar studies a 16.1-fold increase of hardwood KL with *Myceliophthora thermophila* laccase (MtL), a low redox potential fungal laccase, at pH 10 and even a 20-fold increase in molecular weight under the therein determined optimal reaction conditions were achieved by Gouveia et al. [30,31]. Wang et al. achieved a 13.1-fold increase in molecular weight for hardwood and a 4.6-fold increase for softwood KL upon sequential solvent fractionation and polymerization thereof in an aqueous alkaline solution with a genetically modified laccase of bacterial origin [34]. Thus, the herein achieved increases in molecular weight of 6.0-fold for softwood and 19.2-fold for hardwood KL are in accordance with literature.

**Figure 5:**
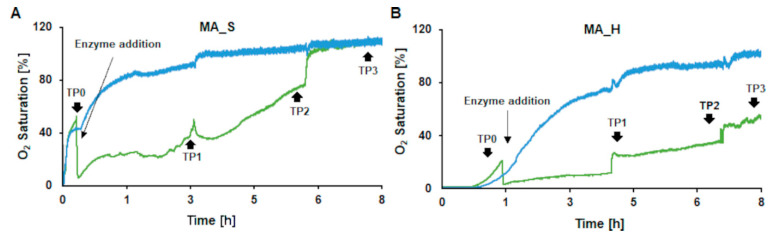
Oxygen consumption during enzymatic oxidation of medium ash softwood (MA_S) (**A**) and medium ash hardwood (MA_H) (**B**) KL samples (green) with CotA compared to a control (blue) without enzyme. The reactions were done at pH 9 with 1 U/mL CotA (for the samples) at 30 °C and with a steady oxygen supply. The timepoints of enzyme addition and sampling (TP0 to TP3) are indicated.

In order to monitor enzyme activity during KL oxidation at a pH of 9, oxygen saturation was followed throughout the reaction and compared to a control sample (without enzyme, but with aeration applied). For the MA_H control, a longer lag time was seen, until the oxygen level started to rise, when compared to MA_S. This may be due to some autoxidation reactions of the dissolved KL. For both samples, a fast decrease in oxygen was observed immediately after the laccase was added to the reaction mixture followed by a slight but steady increase in oxygen saturation. This trend, although with a faster increase in oxygen saturation, was also found for LS oxidation by Ortner et al. [37]. Overall, the final oxygen saturation was lower for MA_H indicating that the enzyme is still active, which may also be linked to the higher concentration of phenol groups present in the hardwood KL. In contrast to that, the phenol content did not significantly change anymore after this incubation time (Figure 4A). Hence, alternative explanations for ongoing oxygen consumption could be potential degradation and polymerization reactions reaching an equilibrium. In any case, oxygen consumption of the samples compared to the control even after 8 h of incubation clearly confirmed that the laccase was still active at pH 9 in the reaction mixture. The stepwise increase in oxygen saturation after each time point of sampling (TP1 to TP3) was generated by the decrease in volume caused by sampling (Figure 5A,B).

In general, the unfractionated hardwood samples were found to be more easily oxidized by the CotA laccase than the fractionated hardwood or softwood samples. The best results were achieved for unfractionated MA_H, which could be explained by the low initial molecular weight and consequently a high number of available free phenol groups. Although the S/G ratio for the hardwoods is naturally higher when compared to the softwoods, this particular sample showed a rather low ratio among the hardwood samples which would point out a higher reactivity. Further, it is known that softwoods generally show a higher degree of condensation than hardwoods, resulting in lower reactivity. MA_H was precipitated at a low pH of 3 and thus it is of high purity, has a high phenol content and a high prevalence of reactive ether bonds in its structure. All these factors lead to the observed high reactivity for the unfractionated MA_H sample. Although membrane filtration leads to a lower ash concentration and a more homogeneous molecular mass distribution in the isolated KL compared to acid precipitation [63], further fractionation of the already acid precipitated samples by membrane filtration did not improve the results herein. Thus, it can be concluded that acid precipitation of KL is sufficient to receive fractions of KL suitable for enzymatic oxidation. Only if the lignin is precipitated at high pH and thus contains a high concentration of ash, further purification would lead to an increase in reactivity as was seen by HA_H KL.

Similar observations were made also in comparable studies. Where KL isolated from different sources (hardwood, softwood or mixtures thereof) and fractionated either by solvent or acid treatment were compared for their susceptibility to enzymatic oxidation. These studies also found that better results were achieved with hardwood than with softwood KL and that higher purified fractions showed a higher reactivity [30,31,32,34].

### 2.3. Comparison of Enzymatic Oxidation of Lignosulfonate and Kraft Lignin

The results for the enzymatic oxidation of KL were compared with those of LS. As elaborated above, it should be noted that enzymatic polymerization of KL and LS was carried out at different pH values and consequently with different enzymes working at the desired reaction pH. Hence, for polymerization of LS, which is possible at pH 7 due to the water-solubility, a laccase originating from the thermophilic fungus *Myceliophthora thermophila* (MtL) was used [64].

**Figure 6:**
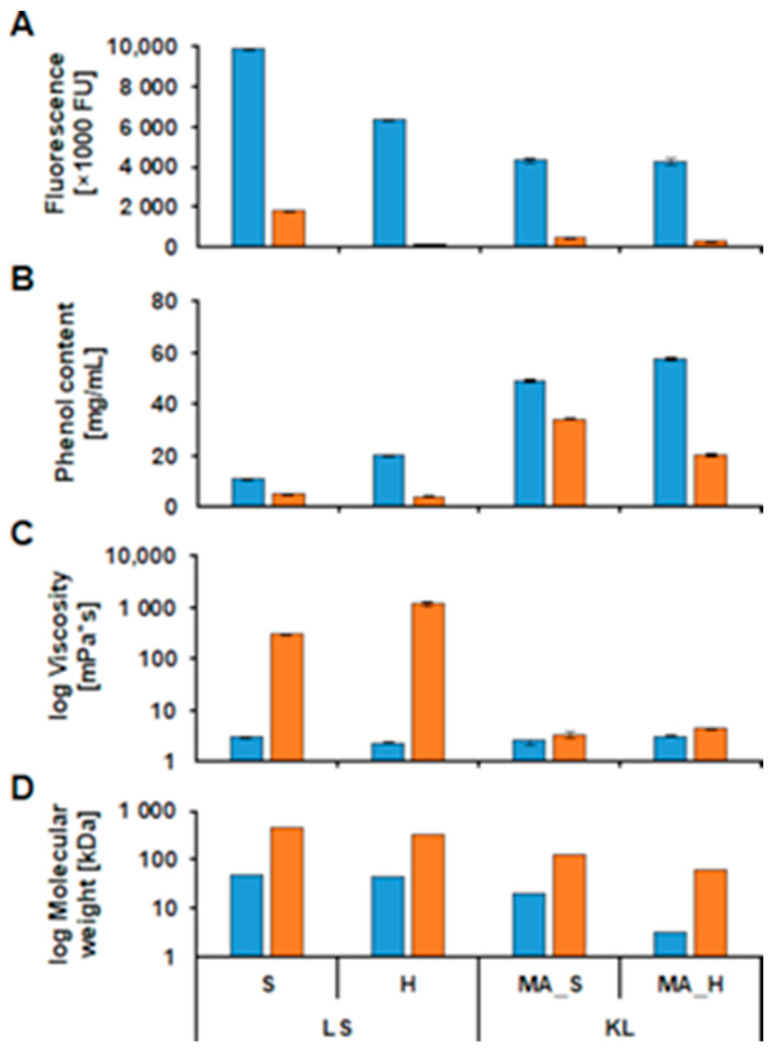
Comparison of fluorescence (**A**), phenol content (**B**), viscosity (**C**) and molecular weight (**D**) for the enzyme catalyzed oxidation of softwood (S) and hardwood (H) lignosulfonates (LS) and medium ash (MA) softwood (S) and hardwood (H) KL. The start (blue bars) and end (orange bars) values of the reaction are compared. LS process was oxidized with the laccase MtL at pH 7 while the KL was oxidized with CotA at pH 9. Both processes were done with a steady oxygen supply. The viscosity and molecular weight data are presented on a logarithmic scale for better comparability.

In general, the hardwood (LS_H) and softwood (LS_S) LS samples showed higher initial fluorescence and molecular weights, the same initial viscosity but a lower initial phenol content when compared to MA_H and MA_S KL. Both types of lignin showed an average decrease of 90% in fluorescence (Figure 6A) upon enzymatic oxidation. In addition, in terms of phenol content, an average decrease of 70% for LS and 50% for KL was observed (Figure 6B). The lowest decrease with only 30% in phenol content was observed for MA_S KL. As for viscosity, enzymatic oxidation caused a dramatically higher increase for the LS samples when compared to KL (Figure 6C). Concerning molecular weight, similar increases between 6.0 to 9.5-fold were found for the samples with higher starting molecular weight (S_LS, H_LS and MA_S KL), while a much stronger increase of up to 19.2-fold was seen for the low-molecular-weight MA_H KL (Figure 6D). The fact that KL showed a higher initial phenol content and fluorescence than LS was expected and originates in the differences between the sulfite and the kraft pulping processes. Since LS molecules generally are of higher initial molecular weight than KL molecules, they are also more likely to reach a higher viscosity at the end of enzymatic oxidation. The yet bigger increase in molecular weight for MA_H can be explained by its lower initial molecular weight leading to better accessibility to the reactive phenol groups, compared to the other samples with higher initial molecular weights. However, the choice of what type of technical lignin to use depends on the intended application of the polymerized products.

## 3. Materials and Methods

### 3.1. Materials

All used chemicals were purchased from Sigma-Aldrich (Steinheim, Germany) or Merck (Darmstadt, Germany) and were of analytical grade.

The hardwood and softwood KL samples used herein were kindly provided by our company partners and were of varying purity, e.g., ash contents (Table 1). The low ash content softwood KL (LA_S) corresponds to Indulin AT, which is a commercially available isolated KL. The medium and high ash content softwood samples (MA_S and HA_S) were acidified black liquors treated with CO_2_ until the respective pH was reached, followed by drying. The same applies to the low and high ash content hardwood samples (LA_H and HA_H). Whereas the medium ash content hardwood sample (MA_H) was received as black liquor and acidified using a 2 N H_2_SO_4_ under stirring at room temperature until a pH of 2 was reached. After filtration, the precipitated KL was re-dissolved and washed, followed by adjusting the pH to 3 through several rounds of washing with water. Afterwards, the filter cake was oven-dried at 70 °C and milled. All the other samples were received as dried filter cakes and were also milled for further use.

The hardwood and softwood LS used herein were kindly provided by Sappi. They originate from used liquor, generated during the sulfite wood pulping process.

### 3.2. Expression and Purification of the CotA Enzyme

The herein used CotA laccase was expressed in competent *E. coli* cells. An expression vector (pET26b(+)) containing a codon-optimized version of the *B. subtilis* CotA encoding gene (Uniprot P07788), an antibiotic resistance marker (Kanamycin), an inducible promoter (T7) and an N-terminal Strep-tagII (GenScript, Piscataway, NJ, USA) was transformed into chemically competent *Escherichia coli* BL21-Gold(DE3) cells (Invitrogen, Carlsbad, CA, USA). The expression of the CotA gene was induced upon IPTG addition. The freshly transformed cells were cultured overnight at 37 °C and 150 rpm (HT Multitron Pro, Infors, Bottmingen, Switzerland) in LB medium (Roth, Germany) containing 40 µg/mL Kanamycin. This overnight culture acted as inoculum for shake flask cultures with a starting OD_600_ of 0.1 where the cells were grown aerobically at 30 °C and 120 rpm until an OD_600_ of 0.6 was reached. At this time point, the incubation temperature was reduced to 25 °C followed by the addition of 0.5 mM IPTG for induction and 0.25 mM CuCl_2_ to the culture medium. After another 4 h of aerobic incubation, the shaking function of the incubator was turned off to achieve microaerobic conditions. After 20 h of incubation under microaerobic conditions, the cells were harvested by centrifugation at 3700 rpm and 4 °C for 20 min (Eppendorf centrifuge 5920 R, Hamburg, Germany). One g cell pellet was resuspended in 5 mL buffer W (100 mM Tris/HCl buffer pH 8.0 with 150 mM NaCl) and disrupted by sonication (Digital sonifier, Branson, CT, USA). The resulting lysate was centrifuged again and sterile filtrated.

Purification of the CotA laccase was carried out by affinity chromatography (Äkta pure, GE Healthcare, Chalfont St. Gilles, UK) according to manufacturer’s protocol (IBA GmbH, Göttingen, Germany). The cleared lysate (5 mL) was loaded onto a Strep-TactinXT cartridge (1 mL). The column was washed with buffer W (1 mL/min) until the baseline was reached. Elution of the enzyme was done during a 3-column volume gradient from 0 to 100% of buffer BXT (100 mM Tris/HCl pH 8.0 with 150 mM NaCl and 50 mM Biotin). PD-10 desalting columns (GE Healthcare, Chalfont St. Giles, UK) were used for a buffer exchange to 100 mM Tris/HCl pH 7.0. The enzyme was stored at −20 °C and 2 mg/mL Bovine Serum Albumin (BSA) was added to increase the storage stability of the enzyme.

### 3.3. Laccase Activity Assay

Laccase activity was assayed by monitoring the oxidation of 2,2′-azino-bis(3-ethylbenzothiazoline-6-sulfonic acid) diammonium salt (ABTS) to its cation radical. The resulting reaction product was measured at 420 nm and measured at a plate reader (Tecan, Infinite M200, Männedorf, Switzerland) [65]. For enzyme dilution, a 0.1 M sodium phosphate buffer with a pH of 7 was used. For the reaction, 170 µL of the diluted enzyme was mixed with 50 µL of a 0.1 M ABTS solution and measured. The activity was expressed in Unit (defined as the amount of enzyme necessary to convert 1 µmol substrate per 1 min). For the reaction, the volumetric activity in U/mL was calculated. All samples were measured in triplicates for statistical analysis. Mean values and standard deviations were calculated.

### 3.4. Enzymatic Oxidation of Various Lignins

Laccase mediated oxidation was carried out in a 50 mL glass bottle containing 30 mL of a 10% (*w*/*v*) solution of the respective KL. The respective sample was suspended in water and the pH of the solution was set to 9, using a 2 N NaOH and stirred overnight at room temperature. Before the reaction was started the pH was re-adjusted and the reaction mixture was aerated and stirred at 500 rpm. At a certain aeration level and the respective reaction temperatures, the reaction was started by the addition of 1 U/mL of the CotA enzyme. The consumption of dissolved oxygen by the enzyme was monitored using a Firesting O_2_ device (Pyroscience GmbH, Aachen, Germany). All experiments were compared to blanks, which contained the respective KL but no enzyme and were otherwise treated like the samples. In order to monitor the progress of the reaction, every 3 h samples were drawn.

The reaction conditions for the enzymatic oxidation of LS were chosen as described in one of our previous studies [17]. Due to a lower pH suitable for the polymerization of LS a laccase originating from the thermophilic fungus *Myceliophthora thermophila* (MtL) [64] was used.

### 3.5. Determination of Phenol Content

The content of phenolic groups was determined using the Folin-Ciocalteu (FC) method. The FC-reagent reacts with phenol groups and forms a blue phosphotungstic-phosphomolybdenum complex that can be quantified by visible light spectrophotometry at 760 nm [66]. The reaction mixture containing 20 µL lignin and 60 µL FC-reagent was filled up to a final volume of 680 µL in MQ-water and incubated for 5 min at 21 °C. Afterwards, 200 µL sodium carbonate 20% (*w*/*v*) and 120 µL MQ-water were added. The samples were incubated for 2 h at 800 rpm and 21 °C. After incubation, 200 µL of the treated samples were transferred into a 96-well plate and the absorbance was measured at 760 nm with the plate reader (Tecan Infinite M200, Männedorf, Switzerland). The measured blank contained MQ-water instead of lignin and was treated like the sample. The phenol concentration in the lignin samples was calculated using vanillin as standard in concentrations ranging from 0.05 to 1.00 mg/mL. All samples were measured in triplicates for statistical analysis. Mean values and standard deviations were calculated.

### 3.6. FTIR Measurement for Determination of S/G Ratio

The S/G ratio of the received KL samples was determined by Fourier Transform Infrared (FTIR) spectroscopy. Small amounts of the milled samples were directly applied onto the ATR-FTIR device (Spectrum 100, PerkinElmer, Waltham, MA, USA).

The FTIR spectrum was recorded in a range from 4000 to 600 cm^−1^ with a resolution of 4 cm^−1^ and 30 scans. The spectra were baseline corrected and normalized in the region from 1600 to 1000 cm^−1^ representing the fingerprint region of KL. All samples were measured in triplicates and the results were averaged to obtain the respective spectrum. The full FTIR spectra of all KL samples can be found in the Supplementary Material (Figure S1).

In the FTIR spectrum of lignin, the band at 1327 cm^−1^ is assigned to the vibration of the S ring and the G ring vibration is found at 1262 cm^−1^. By dividing the respective absorbance (A_1327_/A_1262_) the S/G ratio was calculated [67,68,69]. The calculation of the S/G ratios for the KL samples is shown in the Supplementary Material (Table S1).

### 3.7. Fluorescence Measurement

The fluorescence intensity during the oxidation of lignin was measured as described by Nugroho Prasetyo et al. [70]. One hundred (100) µL lignin sample was mixed with 120 µL of a water solution of methoxyethanol (2:1 *v*/*v*) in a 96-well plate. The sample was excited at 355 nm and the emitted light was measured at 400 nm. The fluorescence was measured with a plate reader (Tecan Infinite M200, Männedorf, Switzerland). All samples were measured in triplicates for statistical analysis. Mean values and standard deviations were calculated.

### 3.8. Viscosity Measurement

Viscosity was followed during the incubation period as a fast, indirect way to determine the increase in molecular weight. For the measurement, 700 µL of the sample were applied to the Rheometer (MCR 302, Anton Paar, Graz, Austria) equipped with a measuring system consisting of a cone plate with a diameter of 50 mm and an angle of 1° (CP50-1). The viscosity measurement was done for 10 s with measuring points made every second at a constant temperature of 20 °C and a constant shear rate of 200 s^−1^. Data analyses were performed with the Anton Paar software RheoCompass 1.24. All samples were measured in duplicates for statistical analysis. Mean values and standard deviations were calculated.

### 3.9. Fractionation of the Received KL Samples

The KL samples were ultrafiltrated using two filtration membranes, differing in their molecular weight cut-off (MWCO), 10 and 5 kDa, respectively. A solution containing 10% (*w*/*v*) of dry substance (DS) of the respective KL sample was set to pH 9, dissolved overnight and filtered through a crossflow Vivaflow 50 R regenerated cellulose membrane (Sartorius, Goettingen, Germany). The membrane was connected to a pump allowing the filtration to be done at a pressure between 2.0 to 2.5 bar. The MWCO used for filtration depended on the initial molecular weight of the samples. For the samples with higher initial molecular weights (MA_S, HA_S and HA_H), the used membrane had a MWCO of 10 kDa, while for the lower molecular weight samples (LA_H and MA_H) the MWCO of the used membrane was 5 kDa (Table 1). After about 5 h the filtration was stopped. At this time point, more than half of the solution has passed through the membrane and was caught in the filtrate where predominantly molecules below the respective MWCO were accumulated (filtrate fraction). The remaining solution in the beaker was enriched in molecules above the respective MWCO (retentate fraction).

### 3.10. Monitoring Changes in Molecular Weight Using Size Exclusion Chromatography (SEC)

SEC analysis was performed by using an Ultimate 3000 autosampler, column oven, UV detector (all Thermo Fisher Scientific Inc., Waltham, MA, USA) equipped with a Dionex HPLC Pump Series P580 (Dionex Softron GmbH, Germering, Germany), Dawn HELEOS I MALLS detectors with lasers operating at either 658 or 785 nm, and an Optilab T-rEX differential refractive index detector, l = 633 nm (all Wyatt Technology, Santa Barbara, CA, USA). Both MALLS detectors were equipped with 18 photodiodes at different measuring angles, with narrow bandpass filters (±10 nm for the respective wavelength used, installed on every second photodiode). The separation was performed with an Agilent PolarGel M guard column (7.5 V × 50 mm) and three PolarGel M columns 7.5 V × 300 mm (5 mm particle size). The columns were kept at 35 °C. The flow rate of the SEC system was set to 0.5 mL with an injection volume of 10 µL and a run time of 65 min per sample. The samples were diluted to a concentration of 1 mg/mL in the mobile phase, which was dimethylsulfoxide (DMSO) containing 0.1% LiCl. Data analyses were done using the ASTRA 6.1 software from Wyatt Technologies.

## 4. Conclusions

In this study, the ability of the alkaliphilic CotA laccase of bacterial origin to oxidize and subsequently polymerize diverse kraft lignins (KL) at alkaline conditions, with a steady oxygen supply was investigated. It was found that the origin and purity of the used KL plays an important role in reactivity. KL isolated from hardwood and with medium purity (MA_H) was found to be more reactive than the less purified hardwood and softwood KL in general. The most reactive softwood KL (MA_S) showed a 6.0-fold increase in molecular weight, while a nearly 20-fold increase was achieved with the most reactive hardwood KL (MA_H), under the herein tested conditions. Finally, a comparison of these results with enzyme-catalyzed polymerization of lignosulfonates (LS), where a fungal laccase was used, showed that LS can be enzymatically polymerized to higher final molecular weights and viscosities but the biggest increase in molecular weight was achieved with KL of high purity (MA_H). With the found reactivity of the CotA laccase and the adapted process set-up for KL, it would be interesting to investigate the enzymatically catalyzed coupling of functional phenolic molecules onto KL.

## Figures and Tables

**Table 1:** Characterization of KL samples.

	LA_S	MA_S	HA_S	LA_H	MA_H	HA_H
**Wood species**	Softwood			Hardwood		
**Ash content**	Low	Medium	High	Low	Medium	High
**pH**	2	2.3	6	2.3	3	8
**S/G ratio**	0.3	0.35	0.34	1.05	1.05	1.38
**Mn [kDa]**	3.0	4.2	4.1	1.5	0.9	2.4
**Mw [kDa]**	12.2	20.6	16.7	4.4	3.2	11.1
**Ð (Mw/Mn)**	4	4.9	4	2.9	3.6	4.5
**Phenol [mg/mL]**	33.21	49.20	31.65	70.04	57.70	26.05

LA_S—Low Ash Softwood (Indulin AT), MA_S—Medium Ash Softwood, HA_S—High Ash Softwood, LA_H—Low Ash Hardwood, MA_H—Medium Ash Hardwood, HA_H—High Ash Hardwood.

## Data Availability

Not applicable.

## Supplementary Materials

The following are available online at https://www.mdpi.com/article/10.3390/ijms222313161/s1.
