# Supplementary material for: Oxidation of Various Kraft Lignins with a Bacterial Laccase Enzyme
Source: Int J Mol Sci. 2021 Dec 6;22(23):13161. doi: 10.3390/ijms222313161 (PMC8658217; doi:10.3390/ijms222313161)
Supplement: Supplementary file 1 [file ijms-22-13161-s001.zip › ijms-1492679-supplementary.pdf]

Supplementary Material for

Oxidation of Various Kraft Lignins with a Bacterial Laccase

Sebastian A. Mayr[1], Raditya Subagia[2], Renate Weiss[1]*, Nikolaus Schwaiger[3], Hedda K. Weber[3], Johannes Leitner[4], Doris Ribitsch[1, 2], Gibson S. Nyanhongo[1, 2], Georg M. Guebitz[1, 2]

[1]Institute of Environmental Biotechnology, University of Natural Resources and Life Sciences (BOKU), Konrad Lorenz Strasse 20, Tulln 3430, Austria; sebastian.mayr@boku.ac.at (S.A.M.); doris.ribitsch@boku.ac.at (D.R.); g.nyanhongo@boku.ac.at (G.S.N.); guebitz@boku.ac.at (G.M.G.)
[2]Austrian Centre for Industrial Biotechnology (ACIB), Konrad Lorenz Strasse 20, Tulln 3430, Austria; radityasubagia@acib.at
[3]Sappi paper holding GmbH, Brucker Strasse 21, Gratkorn 8101, Austria; Nikolaus.Schwaiger@sappi.com (N.S.); hedda.weber@sappi.com (H.K.W.)
[4]Mondi Aktiengesellschaft, Marxergasse 4A, Vienna 1030, Austria; johannes.Leitner@mondigroup.com
*Correspondence: renate.weiss@boku.ac.at; Tel.: +43-1-47654-97486

FTIR spectra of the kraft lignin (KL) samples and determination of S/G ratio

The samples were measured in triplicates and the results were averaged to show the respective spectrum.

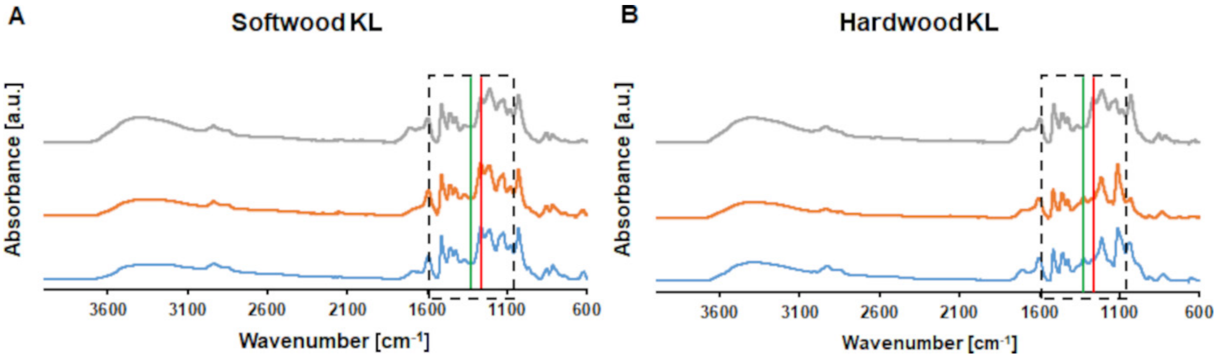

Figure S1. FTIR spectra of the Softwood KL (A) and Hardwood KL (B). The low ash KLs (LA) are shown in blue, the medium ash KLs (MA) in orange and the high ash KLs (HA) in grey, respectively. The vertical green line shows the wavenumber for S (1327 cm^{-1}), the vertical red line the wavenumber for G (1262 cm^{-1}). The dashed black box shows the region of normalization in the range from 1600 to 1000 cm^{-1}.

By taking the absorbance values found at 1327 cm^{-1} (Syringyl) and 1262 cm^{-1} (Guaiacyl) and dividing them, the S/G ratio is determined.

Table S1. Calculation of the S/G ratio

	S (A_{1327})	G (A_{1262})	S/G
LA_S	1.48	4.96	0.30
MA_S	1.65	4.91	0.34
HA_S	1.46	4.14	0.35
LA_H	2.09	1.98	1.05
MA_H	1.88	1.80	1.05
HA_H	2.95	2.13	1.38
